# Population Dynamics and Insecticide Susceptibility of *Anopheles culicifacies* in Malaria Endemic Districts of Chhattisgarh, India

**DOI:** 10.3390/insects12040284

**Published:** 2021-03-25

**Authors:** Tazeen Iram Kareemi, Jitendra K. Nirankar, Ashok K. Mishra, Sunil K. Chand, Gyan Chand, Anup K. Vishwakarma, Archana Tiwari, Praveen K. Bharti

**Affiliations:** 1 ICMR-National Institute of Research in Tribal Health, Jabalpur 482003, India; irumkareemi@gmail.com (T.I.K.); j19nirankar@gmail.com (J.K.N.); akmishra05@gmail.com (A.K.M.); gyanchand_ggg@rediffmail.com (G.C.); anoop.vish0007@gmail.com (A.K.V.); 2 Rajeev Gandhi Technical University, Bhopal, Madhya Pradesh 462033, India; archanargpv@gmail.com; 3 Field Unit, ICMR-National Institute of Malaria Research, Nagpur Road, Garha Jabalpur, Madhya Pradesh 482003, India; skmrc2001@yahoo.co.in

**Keywords:** bionomics, sibling species, CDC light traps, zoophilic, vectorial capacity

## Abstract

**Simple Summary:**

Malaria is a complex disease in part due to multiple vectors having different biological characteristics. In India, there are six primary vectors of malaria viz., *Anopheles culicifacies, An. fluviatitlis, An. stephensi, An. subpictus, An. Minimus,* and *An. epiroticus.* All these vectors have different ecological and seasonal distributions, transmission potential, and insecticide susceptibility status. In addition, except *An. stephensi,* all the five vectors comprise species complexes having sibling species which again differ in characteristics. Therefore, it is imperative to know the characteristics of the local vector population when it comes to planning control strategies. We carried out a study in tribal areas of Chhattisgarh state to investigate the seasonal and ecotype-wise distribution, breeding habits, sibling species composition, insecticide susceptibility, and role in the transmission of the local vector population. A high diversity of species was observed with around 16 species of Anopheles. *An. culicifacies* was the most dominant species and also was found to play a role in malaria transmission. The species was found to be resistant to dichlorodiphenyltrichloroethane (DDT) and Malathion, while an increasing trend of pyrethroid resistance was observed at some sites. Overall, our findings provide a picture of the characteristics of the local vector population in malaria-endemic regions.

**Abstract:**

A study was undertaken in the villages of Korea and Bastar district (Chhattisgarh) during the years 2012–2015 to investigate the bionomics of malaria vectors and the prevalence of their sibling species complexes. Entomological surveys carried out every month included indoor resting collections, pyrethrum spray catches, light trap catches, and insecticide susceptibility status of *Anopheles culicifacies* using World Health Organization (WHO) methods. *Anopheles culicifacies* and *Anopheles fluviatilis* species were assayed by polymerase chain reaction (PCR) for the detection of malaria parasite, and sibling species were identified using PCR and DNA sequencing. A total of 13,186 samples of Anopheles comprising 15 species from Bastar and 16 from Korea were collected. *An. Culicifacies* was recorded as the most dominant species and also the only active vector at both sites. This species was found to be resistant to dichlorodiphenyltrichloroethane (DDT) and Malathion, showing signs of emerging resistance against pyrethroids. Among the sibling species of *An. culicifacies*, the group BCE was found in maximum numbers, while sibling species T of the *An. fluviatilis* was recorded to be dominant among its complex. The study provides a comprehensive view of the vector bionomics in the highly malarious regions of India that may have importance in developing vector control strategies.

## 1. Introduction

With malaria elimination on the agenda, there is a fresh momentum in control interventions throughout the Indian sub-continent. The renewed efforts target providing better surveillance and case management systems, improved and appropriate diagnosis, universal coverage of the population at risk with strengthened vector control interventions, and effective antimalarial drugs. However, this ambitious goal is faced with numerous challenges. Looking at the malaria incidence figures, the country accounted for approximately 0.56 million cases [1] and 77 deaths [2] in the year 2019. According to World Health Organization (WHO) [1], India, along with 29 Sub- Saharan African countries, contributes about 95% of the global malaria cases. This burden is contributed to by varied factors, including diverse topography, multiple parasite strains, and numerous species’ disease vectors. An intensified control effort designed to tune up with this diversity is urgently needed. This requires thorough information on the micro epidemiology of the disease, its vectors, and local factors influencing the overall prevalence.

In India, the majority of malaria morbidity and mortality are contributed to by eight states [3]. Chhattisgarh (CG) state stands second in the list with approximately 18% of the malaria burden and the highest number of deaths [2]. Transmission of malaria in this region is mainly due to two vectors, *Anopheles culicifacies* and *An. fluviatilis*. These species, as well as their sibling species differ in distribution, biological characteristics, vectorial capacities, and susceptibility to insecticides [4,5]. For example, *An. culicifacies* sibling species B is a poor vector or a non-vector, while sibling species A, C, D, and E are considered as vectors. Sibling species E is highly anthropophilic, whereas the other four are primarily zoophilic. Of the four-sibling species of the *An. fluviatilis* complex, species S is an efficient vector, and species T and U are poor vectors, while the role of the V form in malaria transmission remains largely unclear [6]. Although these sibling species differ widely in their physiological and behavioral characteristics, morphologically, they are indistinguishable [7]. Furthermore, reports suggest the change in vector behavior at certain places and the ecological succession of species. *An. fluviatilis* T was found to be playing a role in malaria transmission in Central India. Similarly, *An. culicifacies*, a known indoor resting species, was found parasite positive in outdoor collections. Likewise, a shift in resting preference of *An. fluviatilis* from outdoors to indoors was also observed [4]. These changes are of concern since a sustainable control program demands a specific and targeted approach. Hence, a comprehensive assessment of vector ecology and disease transmission is a crucial consideration in malaria control and elimination. Keeping this in view, we studied the bionomics of malaria vectors and their role in malaria transmission in south and north Chhattisgarh, regions that contribute the majority of the state’s malaria burden [8]. The results provide baseline information on vector distribution, their seasonal and ecotype-wise relative abundance, host preference, resting and breeding habits, role in malaria transmission as well as susceptibility status to insecticides in use.

## 2. Material and Methods

### 2.1. Study Sites and Population

The study was conducted in two districts, Bastar and Korea of Chhattisgarh state. Bastar is situated in the southern part of the state, while Korea lies in the northwestern part. Both the districts have dense forest cover, and the climate is tropical, with rainfall in the months of June to September. The seasons are characterized as Spring (February-March), Summer (April-June), Monsoon (July-September), Post Monsoon (October-November), and Winter (December-January) [9]. The district Bastar receives higher average annual rainfall (1323 mm) than Korea (1225 mm) [10].

The districts have undulating terrain. The river Indrawati flows through Bastar, whereas Korea is drained by the Hasdeo, Tej, Gopad, and Gopri rivers. Numerous streams originating from these rivers bifurcate the districts, and their tributaries provide permanent breeding sites for mosquitoes. District Korea comprises vast masses of forests and hills.

The population is mainly tribal. In the Korea district, the tribal population constitutes 46.2% of the total population [11], while in Bastar [12], this proportion is high as 70%. A large number of tribes still live in deep forests that either remains inaccessible or are risky to approach. Bastar and Korea are violence-prone areas and are subjected to Naxalite activities [13]. The economy is mainly agriculture and forest-based. Most of the population work as daily wage laborers in road construction or other casual work. Houses are of the kutcha type with thatched roofs, incomplete walls, poor or no ventilation, and cattle sheds near or within houses. People spend most of their time outside the house and sleep outdoors or in the verandah (walled from three sides and open from one side). Vector control activities in the study area include spraying Alphacypermethrin inside the human dwelling.

### 2.2. Entomological Collections

Monthly longitudinal entomological surveys were carried out for a period of three years (2012–2015) in the study districts Bastar (19°15′ N, 81°40′ E) and Korea (22nd Korea (it, 81st Korea (it). Two Community Health Centers (CHCs) from each district and 4 villages in each CHC were selected in different geographical ecotypes, i.e., forest, foothill, and plains. Villages located inside deep forests, in foothills (area surrounded by hills or mountains from at least 3 sides), and plains (without forest or hills) were selected to study the difference in vector bionomics in different ecotypes. Villages in the forest ecotype have scattered houses of kutcha type with poor ventilation. Accessibility to roads is very poor, and public transport facilities are also not available. In contrast, villages located in foothill and plain ecotypes have clustered houses and have better accessibility to road and transportation. 

For indoor resting collections, female *Anopheles* mosquito were collected from 2 fixed and 2 randomly selected human dwelling (HD) and cattle sheds (CS) located in different parts of the village using flashlight and mouth aspirators during early morning hours (0600 to 0800) for 15 min in each place as per standard WHO technique [14]. Pyrethrum spray sheet collections (PSSC) were made once a month from human dwellings (HD) randomly selected other than those selected for indoor resting collection during daytime (0600–0010) after knockdown on white sheets by space spraying of a pyrethrum solution as per WHO techniques. Center for disease control (CDC) light traps were used for indoor and outdoor collections from dawn to dusk from 1800 to 0600. Traps were hung at a fixed height of 5.5 feet indoors inside houses and outdoors near the houses and were manually emptied at hourly intervals. The collected adult female mosquitoes were assigned unique codes and stored in dried silica. 

Monthly larval collections were made at all potential breeding sites, including streams, pools, tanks, irrigation fields, and ponds 500 meters around the village and inside the study villages using the standard dip technique (WHO). The larvae were reared in the lab for morphological identification. The abdomen of the blood-fed mosquito was crushed on Whatman filter paper for blood meal analysis. Insecticide susceptibility status of the field caught indoor resting adult *Anopheles culicifacies* mosquitoes was determined against diagnostic dosages of dichlorodiphenyltrichloroethane (DDT) 4%, Malathion 5%, Deltamethrin 0.05%, and Alphacypermethrin 0.05% following the WHO standard procedure [15]. Eight replicates, each with 15 mosquitoes, were used for each insecticide (total 120 mosquitoes for each insecticide). A tube consisting of oil-impregnated paper served as the test control. Mosquitoes were exposed to insecticides for one hour and then transferred to holding tubes for 24 h. The number of dead and alive mosquitoes was counted. The observed and corrected mortality was then calculated as

$$Observed\;mortality=\frac{Total\;number\;of\;dead\;mosquitoes}{Total\;sample\;size}\times 100\;$$

$$Corrected\;mortality=\frac{\left(\%\;observed\;mortality-\%\;control\;mortality\right)}{\left(100-\%\;control\;mortality\right)}\times 100\;$$

### 2.3. Laboratory Processing of Mosquitoes and Detection of Malaria Parasites

Collected mosquitoes were identified up to species level based on the morphological keys [16], and DNA was isolated using methods described by Coen et al. 1984 [17]. The samples were tested for the presence of malaria parasites by polymerase chain reaction (PCR) targeting the 18S rDNA region of *Plasmodium.* The primers and PCR conditions were as per the methodology described by Snounou et al. [18].

### 2.4. Identification of Sibling Species by Molecular Methods

For identification of *An. culicifacies* sibling species complex, D3 and ITS2 regions of ribosomal DNA were amplified and sequenced using methods described by Singh et al. 2004 [19] and Manonmani et al. 2005 [20]. Identification was also made using the methods of Goswami et al. 2006 [21] targeting the COII region of mitochondrial DNA. *An. fluviatilis* sibling species complex was identified using allele-specific PCR described by Singh et al. 2004 [22]. The primers and conditions of PCR were the same as described in [22].

### 2.5. Blood Meal Analysis for Identification of Host Preference of the Vectors

Host preference of mosquitoes was determined by gel diffusion assay against human and bovine anti-sera using the collected blood meals of Anophelines [23]. Briefly, the dried blood spots from the vector abdomen were cut and dissolved in normal saline (0.7 g/100 mL distilled water) for 4.5 h. Fifty milliliters of solution 1v(50% barbiturate buffer pH 8.6, 49.8% distilled water, and 0.2% agarose) was added to 50 mL of solution 2 (0.9% agarose in barbiturate buffer solution). Three milliliters of this mixed solution was spread on micro slides and allowed to solidify. Human and bovine test antisera and mosquito blood dissolved in normal saline were pipetted into the wells in the gel slides. A precipitation line around the wells, formed when serum came into contact with antiserum, indicated a positive result.

### 2.6. Data Analysis

The data were double key-entered in MS Excel 2007 (Microsoft Corporation, Redmond, Washington, DC, USA). Categorical data are presented as percentages (frequency counts) and compared using Pearson’s chi-square test. Continuous data are represented as mean (± standard deviation) and compared using ANOVA (one-way analysis of variance) with Bonferroni adjustment for multiple comparisons. Log transformed data were used in the ANOVA. Statistical analysis was done using STATA software version 8.0 (StataCorp, LP, TX, USA). Per man-hour density (PMHD) for indoor resting collections was calculated by dividing the total mosquito collected by the number of man-hours spent in the collection. The significance of differences between PMHD of two sample sets was calculated using a *t*-test.

## 3. Results:

### 3.1. Species Diversity, Seasonal, and Ecotype-Wise Abundance of Anophelines in the Study Sites

A total of 997 (524 h in Bastar and 473 in Korea) man-hours were spent on indoor resting mosquito collections at both the study sites. The Anopheline fauna consisted of 15 species in Bastar and 16 in Korea, comprising a total of 13,186 mosquitoes collected from both sites. The average per man-hour density of all the collected *Anopheles* species in indoor collections (human dwellings and cattle sheds) was 12.49. (Table 1). The diversity of Anopheles species was almost the same in both the sites, with the difference that *An. stephensi* and *An. jamesi* were collected from Korea and *An. varuna* from Bastar only. Species collected from both the sites (Table 1) included *An. culicifacies*, *An. fluviatilis, An. subpictus*, *An. vagus, An. annularis, An. barbirostris*, *An. splendidus, An. Tessalatus, An. aconitus, An. nigerimus, An. pallidus,* and *An. theobaldi. An. culicifacies* comprised the majority of the collections (38% in Bastar and 46.7% in Korea), having densities as 4.7 (95% CI 3.5–5.7) and 6.2 (95% CI 5.2–7.2) in Bastar and Korea, respectively. The average per man-hour (PMHD) density of *An. culicifacies* was significantly higher in Korea as compared to Bastar (*p* < 0.01). *An. subpictus* (27%) was the second most abundant species in both sites. *An. fluviatilis* was observed in low densities with 0.34 (95% CI 0.14–0.56) and 1.7 (95% CI 1.25–2.15) of PMHD in Bastar and Korea, respectively.

**Figure 1:**
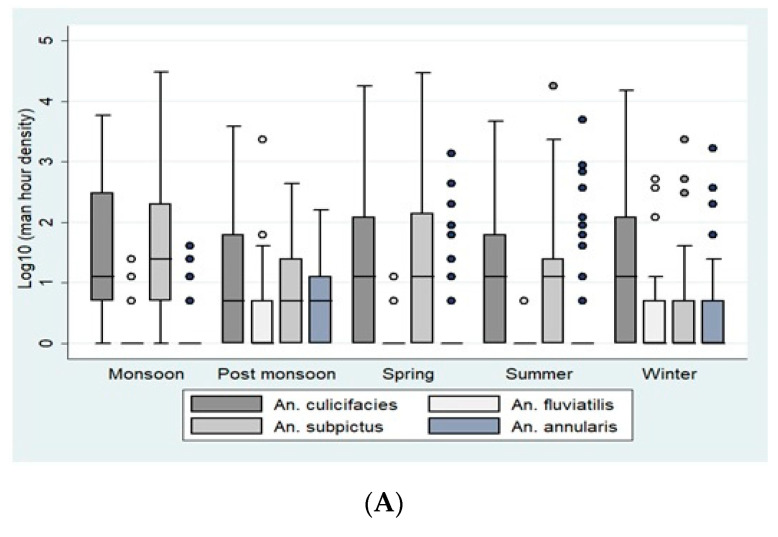

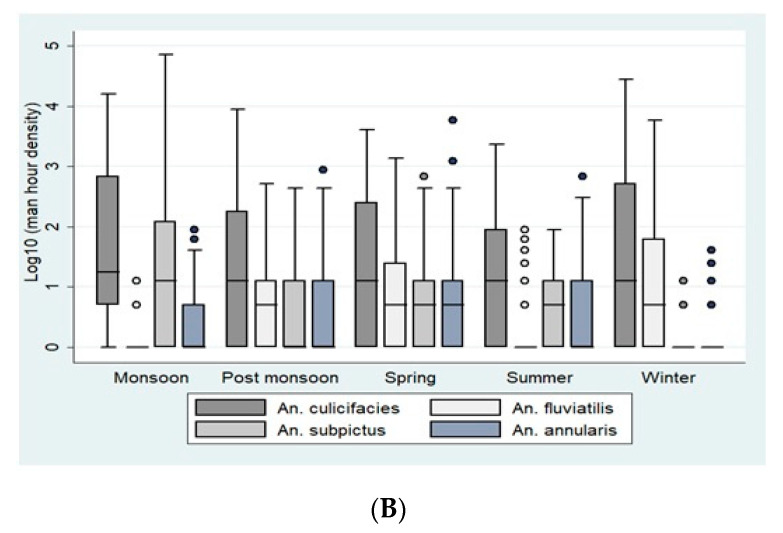
(**A**). Season-wise average per man-hour density (indoor resting collections) in Bastar. (**B**). Season-wise average per man-hour density (indoor resting collections) in Korea district. The boxes in the plots correspond to the log-transformed values of man-hour densities of *An. culicifacies, An. fluviatilis, An. subpictus,* and *An. annularis* across different seasons. The densities are represented in the boxes as Quartile 1, median, and Quartile 3. The whiskers represent the highest and the lowest densities.

A high abundance of *Anopheles* mosquitoes was observed throughout the year showing seasonal peaks and dips (Figure 1A,B). The periods of monsoon and spring showed the highest densities of Anopheline at both sites, with the largest peak observed in the monsoon. The densities of *An. culicifacies* also peaked during monsoon with a PMHD of 8.83 (95% CI 6.7–10.9) in Bastar and 12.67 (95% CI 9.25–16.08) in Korea. However, the species was recorded in large numbers throughout the year. *An. fluviatilis* densities were highest during the winter season, while its abundance was also recorded during monsoon in Bastar and during spring in Korea. In Bastar, the density of *An. fluviatilis* during winter was 3.12 (95% CI 1.08–5.1), and in Korea, it was 6.03 (95% CI 3.1–8.8).

**Figure 2:**
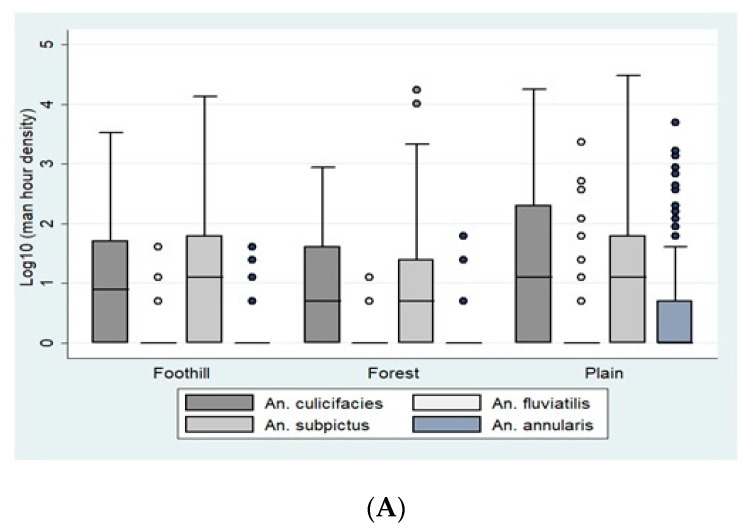

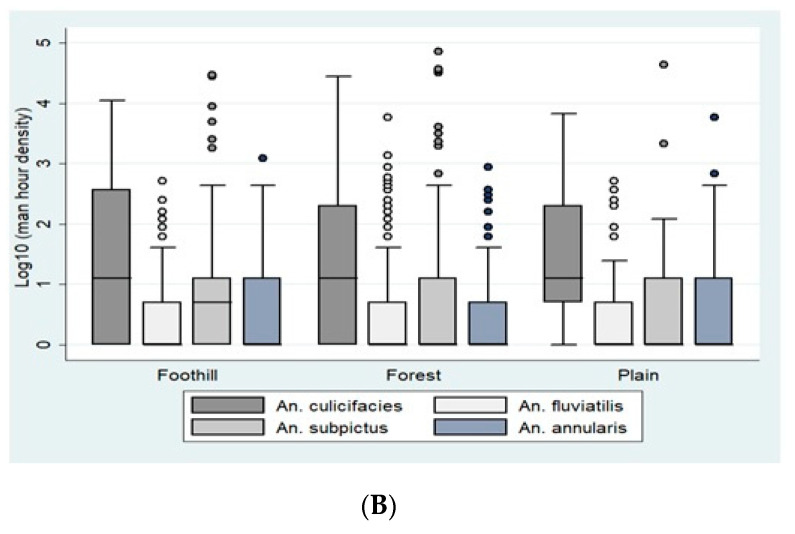
(**A**). Ecotype-wise per man-hour density (Indoor Resting Collection) in district Bastar. (**B**). Ecotype-wise per man-hour density (Indoor Resting Collection) in district Korea. The boxes in the plots correspond to the log-transformed values of man-hour densities of *An. Culicifacies, An. Fluviatilis, An. Subpictus,* and *An. annularis* across different ecotypes, including foothill, forest, and plain. The densities are represented in the boxes as Quartile 1, median, and Quartile 3. The whiskers represent the highest and the lowest densities

Ecotype-wise analysis of Anopheline distribution in Bastar showed that *An. culicifacies* was most abundant in the plains (85%) with a mean density of 9.01 (95% CI 7.5–10.4), while its densities and the density of other Anopheline was low in forest and foothill ecotypes. Maximum numbers of *An. fluviatilis* in Bastar were recorded from the plains (Mean 3.42; 95% CI 1.2–5.5). In the Korea district, the forest ecotype had the maximum density of mosquitoes with *An. culicifacies* being the most abundant. The average PMHD of *An. culicifacies* in the forest ecotype of Korea was 10.2 (95% CI 8.01–12.39), while in the plain, it was 7.81 (95% CI 5.9–9.2) and 9.65 (95% CI 7.6–11.6) in the foothill ecotype. The density of *An. fluviatilis* in this site was higher in forest ecotypes (Mean 5.05; 95% CI 3.3–6.7), comprising 54% of the collections (Figure 2A,B).

**Figure 3:**
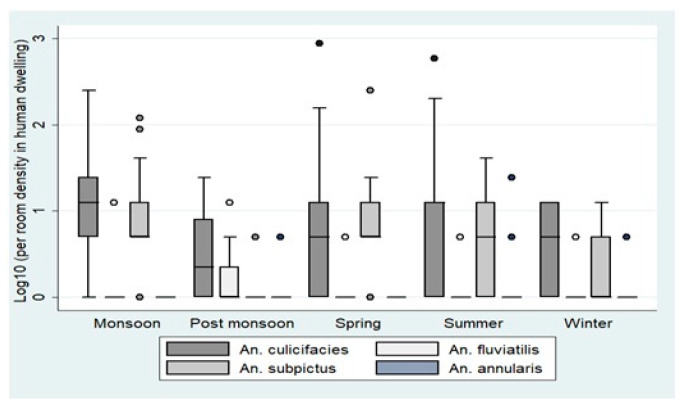
Seasonal pyrethrum spray catches in human dwellings in districts Bastar and Korea. The boxes in the plots correspond to the log-transformed values of per room density in human dwellings across different seasons. The densities are represented as Quartile 1, median, and Quartile 3. The whiskers represent the highest and the lowest densities.

Pyrethrum spray sheet collections conducted in houses at each site recorded 10 *Anopheles* species from Bastar and 8 from Korea. *An. culicifacies* was the most abundant species at both sites. *An. subpictus, An. Vagus,* and *An. annularis* were also collected in large proportions. Other species observed, though in few numbers, included *An. barbirostris* and *An. splendidus,* while *An. tessalatus* and *An. aconitus* were recorded from Bastar and *An. jeyporiensis* from Korea only. The month-wise distribution of *An. culicifacies* showed peaks during March-April and July-August. *An. fluviatilis* was mainly found from November to March (Figure 3).

**Figure 4:**
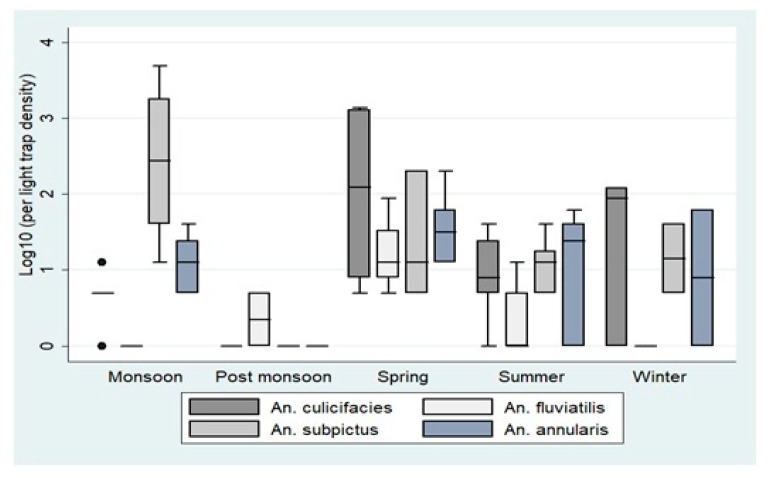
Seasonal light trap catches in districts Bastar and Korea. The boxes in the plots correspond to the log-transformed values per light trap catch density across different seasons. The densities are represented in the boxes as Quartile 1, median, and Quartile 3. The whiskers represent the highest and the lowest densities.

In light trap catches, 12 nights in Bastar and 17 nights in Korea were spent for collections; 71 CDC light traps in the former and 203 in the later site (Figure 4). In Bastar *An. culicifacies, An. subpictus, An. Vagus,* and *An. annularis* were recorded, while in Korea, in addition, *An. fluviatilis* and *An. jeyporiensis* were also caught. *An. subpictus* was the dominant species in night catches at both sites. *An. culicifacies* comprised 19.7% of collections in Bastar and 31.5% in the Korea district. The proportion of *An. fluviatilis* was only 7%. Season-wise distribution studies showed the maximum abundance of *Anopheline* in the monsoon, while the highest night catches of *An. culicifacies* and *An. fluviatilis* were recorded in spring.

### 3.2. Vector Incrimination

A total of 5668 samples of *An. culicifacies* (3103 from Korea and 2565 from Bastar) and 661 *An. fluviatilis* (547 from Korea and 114 from Bastar) were tested for the presence of malaria parasites. A total of five *An. culicifacies* mosquitoes were found infected, three with *Plasmodium falciparum* (Pf) and two with *Plasmodium vivax* (Pv). Both the Pv positive mosquito and two of the Pf positive were collected from Korea. Of these, one Pf positive mosquito was collected in a pyrethrum spray sheet, while the rest of the three from cattle sheds. One notable observation was that all the parasite-positive mosquitoes from Korea were collected in May in two successive years. Only one Pf positive mosquito was from Bastar collected from a cattle shed in the month of March. None of the *An. fluviatilis* was positive for malaria infection (Table 2). All the parasite-infected *An. culicifacies* belonged to the “BCE type” sibling species group.

### 3.3. Sibling Species Distribution in the Study Sites

Sibling species identification was made by PCR and DNA sequencing targeting D3, Internal transcribed spacer (ITS2), and Cytochrome c oxidase subunit 2 (COII) regions. However, we could not identify all five-sibling species of *An. culicifacies* due to the lack of an accurate molecular identification technique. The species was identified into two groups only: A/D and B/C/E. Of 600 samples tested, group B/C/E was the most abundant, comprising 87%. Analysis of *An. fluviatilis* sibling species complex by 28S rDNA allele-specific PCR revealed that sibling species T was dominant (70%) at both sites. Sibling species S comprised only 4% of the total *An. fluviatilis* tested from both Korea and Bastar.

### 3.4. Host Preference of An. culicifacies and An. fluviatilis

Out of 223 *An. culicifacies* and 75 *An. Fluviatilis* blood-fed mosquito tested for host blood meal preference, seven were found having human blood, while remaining 291 were positive with the bovine serum. In Bastar district, five *An. culicifacies* mosquitoes and none of the *An. fluviatilis* were human blood positive. While in the Korea district, one sample of both the vectors was found positive for human blood.

### 3.5. Anopheline Fauna Composition of Breeding Sites

A total of 777 Anopheles mosquito larvae were collected, representing nine species in Bastar and 12 species in the Korea district from potential breeding sites, such as ditches, ponds, river pool, running water, seepage water, stream, pool, sandy pit, rice field, rocky pit, and rock stream. *An. culicifacies* was the most abundant species representing 36% and 45.1% of total collections from Korea and Bastar, respectively. This species was found breeding mainly in streams, seepage water, and rock pits and showed peaks of relative abundance in March (34.8%) and September (13.8%); large numbers of larvae were also obtained in June. In addition, *An. culicifacies*, *An. subpictus* was found in abundance (26% in Bastar and 17.5% in Korea). *An. fluviatilis* occurred in low densities, accounting for 3.1% in Korea and 1.67% in Bastar, and breeding mainly in seepage water, rock pits, and streams from October to April.

### 3.6. Status of Susceptibility to Insecticides in An. culicifacies

Insecticide susceptibility status of *An. culicifacies* was determined in districts Bastar and Korea for two and three successive years, respectively (Table 3). The species was resistant against DDT and Malathion in both the districts with corrected mortality of 6.6 ± 2.8 and 65.8 ± 3.4, respectively. Resistance was recorded against deltamethrin (73.7 ± 6.35) and alpha-cypermethrin (78 ± 5.05) in district Bastar. However, in Korea, the species remained susceptible to alpha-cypermethrin, having a mortality rate of 98.6 ± 0.7, with possible resistance to deltamethrin (91.5 ± 1).

## 4. Discussion

The present study provides information on the bionomics of malaria vectors in different ecotypes through all seasons in two distant districts situated at opposite ends of the malaria-endemic state Chhattisgarh. Studies on vectors in this region have been limited [8]. Our work demonstrated high Anopheline species abundance and diversity in both the study sites. *An. culicifacies* was incriminated as the only vector and was the most abundant species collected throughout the year. *An. fluviatilis* was caught in low densities, contrary to the previous findings where this species was dominant, whereas the former was obtained in few numbers. However, in other parts of Central India, *An. culicifacies* has been documented to be the most prevalent species [24]. Mishra et al. when carried out a study similar to the present one in the forested areas of Central India as a part of the Malaria Elimination demonstration project, *An. culicifacies* was collected in maximum numbers, highest densities recorded in the month of July.

In the present study, the prevalence of *An. culicifacies* showed two peaks, first in February–March and second in August–September, coinciding with the paddy cultivation regimes in the study villages. *An. fluviatilis* densities were recorded in the winter season (November–February). Seasonal distribution of parasite-infected mosquito showed that most of the infected *An. culicifacies* was found in the month of May, i.e., the summer season. Actually, the spray campaigns are organized pre- and post-monsoon (June–July and September–October). As the effectiveness of sprayed insecticides declines, the mosquito density increases. This, along with other contributing factors, may be the reason for high mosquito density and disease transmission during summer. Ecotype-wise analysis of species distribution showed that the density of *An. culicifacies* and other *Anopheles* species were highest in plains in the district Bastar, while in Korea, the forest ecotype had the maximum densities of all the *Anopheline* species. Previous studies undertaken established distinct differences in intensity of malaria transmission in plain and forest ecotypes. Observations similar to ours have been made in Madhya Pradesh [25].

Despite observations made in previous studies [8], no *An. fluviatilis* mosquitoes were found to be infected with the malaria parasite. This requires further investigations as the present findings might be due to the low abundance of the species or due to collection/trapping bias. *An. culicifacies* was the only species recorded to transmit malaria in both sites. However, the infection rates were low despite the fact that the study sites selected are highly malarious. Cases of mixed infections and also a rare case of quadruple infection have been recorded in the periods when infection rates in our collections remained zero. This indicates the existence of high levels of malaria incidences even in the presence of low transmission rates. Similar findings have been made previously [26,27,28]. Our results highlight that probably many malaria cases were due to a single infective bite. This is also suggestive of the outdoor resting habit of vectors due to indoor spraying operations, thus the need for outdoor control measures. A total of 6329 mosquito specimens were tested, and out of this, five were found positive by PCR. This showed that active transmission is going on in this area. Although there are a few glitches reported in the literature, such as the inhibition of PCR results when whole mosquitoes are used for DNA isolation, the removal of the head and thorax seemed to negate the inhibition, suggesting the presence of inhibitors in these body parts. [29].

The study revealed that both *An. culicifacies* and *An. fluviatilis* are strongly zoophilic in the study area. They prefer to rest in cattle sheds that are in the vicinity of human dwellings and are generally dark, damp, and humid. *An. culicifacies* is an established zoophilic vector [8], whereas zoophily in *An. fluviatilis* could be due to the predominance of sibling species T in the area. This kind of behavior in vector species provides them additional benefit as they avoid the insecticide-treated wall surfaces of human dwellings. This also increases the survival probability of infectious mosquitoes as they escape control interventions which somehow pose a threat to vector control activities.

The Vector control program in Chhattisgarh state employs DDT and pyrethroid-based indoor residual spray (IRS) and long-lasting insecticidal nets (LLINs). We evaluated the susceptibility of *An. culicifacies* to insecticides in current use. The species was found to be resistant to DDT and Malathion in both the study sites. There seemed to be an increasing trend of pyrethroid resistance in Bastar, while the vector remained susceptible or possibly resistant in Korea. Studies conducted in other parts of Chhattisgarh state also report resistance against one or more classes of insecticides [30,31]. In the adjoining state Madhya Pradesh, *An. culicifacies* was found to be resistant against DDT, Malathion, Alphacypermethrin with possible resistance against Deltamethrin [24]. Resistance against pyrethroids has been widespread in the states of Odisha, Chhattisgarh, and Madhya Pradesh [5]. With only a few insecticides available presently, the development of resistance against all the categories of insecticides in vectors is of concern. However, the epidemiological impact of insecticide resistance remains largely disputed. Studies report that although vectors have developed resistance to DDT and pyrethroid, the excito-repellant effect of these insecticides provides sufficient protection against mosquito bites. But there are examples of malaria case resurgence due to the development of insecticide resistance in the vector [32,33]. Therefore, there is a compelling need to monitor the effectiveness of control strategies continuously. In addition, detection of genetic markers responsible for resistance in vectors is needed to monitor the spread of resistance.

The findings of the present study provide insight into nearly all the aspects of vector bionomics in the study area. However, there are certain limitations to the study. The habitat parameters of breeding sites could not be determined so as the results generated are informative only of the vector bionomics of the sites. The number of parasite-positive mosquitoes were few. Therefore, the entomological inoculation rate (EIR) could not be calculated. Second, due to the unavailability of the insectary strain of *An. culicifacies*, we had to use field-collected adult mosquitoes of mixed physiological age in the insecticide susceptibility assay. The study site is almost 700 km far from the institute. The emergence of enough larvae from the field and their maintenance was almost impossible in this field setting. Therefore, we used the field-caught mosquitoes for the susceptibility assay irrespective of their age, etc. 

The unreliability of the available molecular techniques for identification of sibling species of *An. culicifacies* proved to be a hurdle in the identification of the species up to the subspecies level. However, the species was accurately grouped into “BCE” and “AD” types. BCE was the dominant type. Nearly 97% of *An. culicifacies* tested were found positive for bovine blood. Sibling species B and C are known to be zoophilic [34], suggesting the predominance of these two-sibling species in the area. All the malaria parasite-positive mosquito belonged to the “BCE type”, indicating the probable role of sibling species C in malaria transmission as B is a poor- or non-vector. Sibling species C is an established vector in other parts of central India [4].

Based on observations made in this study, the following suggestions are put forward for further research and implementation of vector control:

Continuous monitoring of insecticide resistance and elucidation and characterization of the underlying mechanisms in vectors is required to evaluate its impact on control interventions and malaria transmission.

There is a need to strengthen vector control in the study areas. The high densities of vector species despite control interventions point towards the need for studies to evaluate the effectiveness of such measures. The efficacy, household acceptability, and feasibility of LLINs should be assessed. The target dose of the insecticide spray formulation and the residual efficacy of the applied insecticides should be monitored. An appropriate combination of IRS, LLINs, and larval control activities is needed to bring down the vector densities effectively.

Insecticides spray campaigns should be targeted to cover the months of February- April and October–November as malaria transmission occurs during these periods also. The IRS should be directed towards cattle shelters also as the vectors in our study were found to be predominantly zoophilic. 

The bionomics of vector species differs globally. The responses of these vectors to control interventions differ likewise. Similarly, various biological, cultural, and environmental factors regulate the dynamics of disease transmission in an area. Therefore, to have the greatest impacts, the efforts to control should be channeled as per the needs specific to a country, village, or region.

## 5. Conclusions

The present study discusses important aspects of vector bionomics, including the seasonal abundance, host and habitat preferences, insecticide susceptibility, sibling species complexes as well as malaria transmission potentials. The complete Anopheline fauna of the region was studied, and sibling species identified. However, the biological characteristics of individual sibling species, as well as their role in malaria transmission, need to be further assessed to help the policymakers design suitable strategies.

## Figures and Tables

**Table 1:** Total number of *Anopheles* mosquito collected from study sites using different collection methods (Indoor resting, light trap, and pyrethrum spray sheet collections).

Species		Bastar					Korea				Total
	Indoor Resting (HD)	Indoor Resting (CS)	PMHD IRC (HD+ CS)	LT	PSSC	Indoor Resting (HD)	Indoor Resting (CS)	PMHD IRC (HD +CS)	LT	PSSC	
*An. culicifacies*	130	2342	4.7	14	79	126	2798	6.2	66	113	5668
*An. fluviatilis*	1	113	0.2	0	0	16	508	1.1	15	8	661
*An. subpictus*	210	2065	4.3	23	58	70	1212	2.7	101	62	3801
*An. jeyporiensis*	0	3	0.006	0	0	22	568	1.25	1	2	596
*An. varuna*	56	303	0.68	17	30	19	54	0.15	6	29	514
*An. annularis*	13	337	0.67	17	2	21	520	1.14	43	4	957
*Other Anophelines*	27	558	1.11	15	4	7	360	0.77	17	1	989
Total	437	5721	11.7	86	173	281	6020	13.3	249	219	13186

HD: Human dwellings; CS: Cattle sheds; LT: Light trap; PSSC: Pyrethrum Spray sheet collection, PMHD: Per man-hour density, IRC: Indoor Resting Collection.

**Table 2:** Ecotype and season-wise *Anopheles* mosquito diagnosed for malaria parasites (*Plasmodium falciparum* and *P. vivax)* by polymerase chain reaction (PCR) method.

Site	Ecotype	Season	*An. culicifacies* (no. of Mosquito Positive/no. of Mosquito Tested)	*An. fluviatilis* no. of Mosquito Positive/no. of Mosquito Tested)	Total no. of Mosquito Positive/no. of Mosquito Tested)
Bastar	Forest	Summer	0/39	0	0/39
		Monsoon	0/50	0	0/50
		Post Monsoon	0/18	0/3	0/21
		Winter	0/16	0/3	0/19
		Spring	0/58	0	0/58
	Foothill	Summer	0/27	0	0/27
		Monsoon	0/102	0/3	0/105
		Post Monsoon	0/30	0/5	0/35
		Winter	0/14	0/2	0/16
		Spring	0/39	0/2	0/41
	Plain	Summer	0/511	0/2	0/513
		Monsoon	0/528	0/3	0/531
		Post Monsoon	0/228	0/41	0/269
		Winter	0/458	0/45	0/503
		Spring	1/447	0/5	1/452
	**Total (Bastar)**		**1/2565**	**0/114**	**1/2679**
Korea	Forest	Summer	**2/227**	0/21	**2/248**
		Monsoon	0/485	0/4	0/489
		Post Monsoon	0/185	0/50	0/235
		Winter	0/238	0/115	0/353
		Spring	0/362	0/108	0/470
	Foothill	Summer	**2/223**	0/14	**2/237**
		Monsoon	1/298	0/2	0/300
		Post Monsoon	0/141	0/35	0/176
		Winter	0/117	0/43	0/160
		Spring	0/186	0/44	0/230
	Plain	Summer	0/126	0/4	0/130
		Monsoon	0/142	0	0/142
		Post Monsoon	0/119	0/15	0/134
		Winter	0/129	0/23	0/152
		Spring	0/125	0/69	0/194
	**Total (Korea)**		**4/3103**	**0/547**	**4/3650**
**Total (Korea and Bastar)**			**5/5668**	**0/661**	**5/6329**

**Table 3:** Insecticide susceptibility status of *An. culicifacies* against dichlorodiphenyltrichloroethane (DDT), Malathion, Alphacypermethrin, and Deltamethrin in districts Bastar and Korea of Chhattisgarh state during 2014–2016.

**District**	**Year**	**% Mortality (R/S)**			
		**DDT (4%)**	**Malathion (5%)**	**Alphacypermethrin (0.05%)**	**Deltamethrin (0.05%)**
**Bastar**	2014	6.6 (R)	76.2(R)	88.1 (PR)	86.4 (PR)
	2015	Nd	Nd	78(R)	73.7(R)
**Korea**	2014	12.9 (R)	73.4 (R)	100 (S)	92.7 (PR)
	2015	Nd	Nd	98.6 (S)	91.5 (PR)
	2016	15 (R)	65.8(R)	100 (S)	97.4 (S)

R and S refer to resistant and susceptible status of *An. culicifacies*; nd: not done; PR: possible resistance.

## Data Availability

Data that support the finding are contained within the article.
